# Prevalence and Risk Factors for Diabetic Peripheral Neuropathy Among Malagasy Patients With Type 2 Diabetes Mellitus: A Cross‐Sectional Study

**DOI:** 10.1002/hsr2.72209

**Published:** 2026-03-22

**Authors:** Sitraka Angelo Raharinavalona, Naliniaina Robert Randrianatoandro, Rija Eric Raherison, Thierry Razanamparany, Andrianirina Dave Patrick Rakotomalala

**Affiliations:** ^1^ Cardiovascular Diseases and Internal Medicine departments Soavinandriana Hospital Center Antananarivo Madagascar; ^2^ Neurology and psychiatry departments, Soavinandriana Hospital Center Joseph Raseta Befelatanana University Hospital Center Antananarivo Madagascar; ^3^ Endocrinology Department Joseph Raseta Befelatanana University Hospital Center Anta‐nanarivo Madagascar

**Keywords:** diabetic peripheral neuropathies, prevalence, risk factors, type 2 diabetes mellitus

## Abstract

**Background and Aims:**

Diabetic peripheral neuropathy (DPN) is the most prevalent complication of diabetes, leading to functional impairment and a decline in patients' quality of life. The objectives of the present study were to determine the prevalence and risk factors for DPN in Malagasy patients with type 2 diabetes (T2DM).

**Methods:**

A cross‐sectional study was conducted in the Internal Medicine and Cardiovascular Diseases Departments of the Soavinandriana hospital center over a 3‐year period. The diagnosis of NPD was made in the presence of a DN4 questionnaire score ≥ 4, impaired sensitivity to a 10 g monofilament and reduced or absent ankle reflexes.

**Results:**

Among the 289 T2DM, the prevalence of DPN was 28.7%. After multivariate analysis, risk factors for DPN were age ≥ 60 years (OR 2.21 (1.19–4.08)), dyslipidemia (OR 3.38 (1.52–7.52)), microalbuminuria (OR 6.55 (3.69–11. 6)), diabetes duration ≥ 10 years (OR 2.92 (1.54–5.53)), glycated hemoglobin ≥ 7% (OR 2.43 (1.08–5.49)), nephropathy (OR 12.8 (6.71–24.3)) and retinopathy (OR 2.32 (1.25–4.31)).

**Conclusion:**

DPN was a prevalent comorbidity among Malagasy patients with T2DM. The risk factors were consistent with those documented in the literature. The primary strategy for preventing DPN is the effective management of diabetes and modifiable risk factors.

## 1 Introduction

Diabetic neuropathies are a heterogeneous group of disorders affecting different parts of the nervous system and presenting with diverse clinical manifestations [1]. Their pathogenesis involves multiple factors responsible for nerve and vascular toxicity via different mechanisms, including oxidative stress, inflammation, hyperglycemia and dyslipidemia [2, 3, 4].

Diabetic peripheral neuropathy (DPN) has been identified as the most prevalent form of neuropathy, with prevalence estimates ranging from 6% to 51% among adult populations worldwide [5, 6]. These conditions are frequently under‐diagnosed and insufficiently treated [7]. However, DPN can result in a range of adverse outcomes, including painful symptoms, negative motor symptoms, lower‐limb amputations, and physical disability, consequently leading to a reduced quality of life. Additionally, they are associated with an increased all‐cause mortality [8, 9]. Risk factors for DPN are advanced age, diabetes duration, poor glycemic control, dyslipidemia, body mass index, and presence of other microvascular complications [10, 11].

In Africa, the overall prevalence of DPN was 46%, with the highest prevalence recorded in West Africa (49.4%) and the lowest in Central Africa (35.9%) [12]. A paucity of studies has been conducted on this subject in Madagascar. It is hypothesized that DPN are prevalent among the Malagasy diabetic population and is associated with specific risk factors. The objectives of the present study were to determine the prevalence of DPN and their risk factors in Malagasy patients with type 2 diabetes mellitus (T2DM) seen at the Soavinandriana hospital center.

## 2 Methods

### 2.1 Study Design and Setting

A cross‐sectional study was conducted in the Internal Medicine and Cardiovascular Diseases Departments of the Center Hospitalier de Soavinandrina in Antananarivo, Madagascar's capital. These departments are responsible for the management of all cardiovascular, internal medicine, endocrine, and metabolic diseases. On average, 800 patients are hospitalized and 5400 are followed up on an outpatient basis each year, of whom approximately 650 are diabetics. The study was conducted over a period of 3 years, from January 1, 2021, to December 31, 2023.

### 2.2 Study Population

Our study population consisted of T2DM patients who had been screened for DPN. We included all known or newly diagnosed T2DM patients, with or without DPN, followed as outpatients at our study site. Patients with chronic alcoholism, undergoing chemotherapy or radiotherapy, with a neurological or mental disorder that made it difficult for them to answer the questionnaire, or those with incomplete records, were excluded.

The sampling method employed was a simple random sample without replacement, and the sample size was calculated using the following formula: *n* = (*Z*1 − *α*/2)² *P* (1 − *P*)/*d*², based on the assumptions of a 95% confidence level (*Z*1 − *α*/2 = 1.96), a 5% margin of error, and a 26.76% prevalence of DPN [13]. The sample size was estimated at 302 patients with T2DM.

### 2.3 Study Variables

The following variables were studied: socio‐demographic data (age, sex), cardiovascular risk factors (hypertension, dyslipidemia, microalbuminuria, current smoking, overweight or obesity, menopause), and T2DM (duration, glycated hemoglobin or Hb A1c, retinopathy, nephropathy), previous medication (antidiabetics, antihypertensives, statins, antiplatelet agents), and DPN (symptoms, DN4 questionnaire, foot deformity, monofilament testing, ankle reflexes, symptomatic medication).

Diabetes was diagnosed and classified according to the criteria of the American Diabetes Association [14] or if antidiabetic medication was used. The DN4 questionnaire was completed prior to each test. The 10 g Nylon® 5.07 gauge monofilament test is performed in a quiet, relaxed environment. The monofilament is applied to the patient's hands so that they know what to expect. Patients are asked to close their eyes so they cannot see where the monofilament is being applied. The monofilament should be applied perpendicular to the skin surface with enough force to bend it. The total application time should be one and a half seconds. Test 3 plantar sites on both feet: opposite the ball of the first toe and the heads of the first and fifth metatarsals. The monofilament should be applied firmly, all at once, taking care not to slide along the skin, not to touch the skin repeatedly, and not to create calluses or ulcers. Sites are tested one at a time, in no particular order, to avoid bias due to patient anticipation. For each site, the patient is asked to say “yes” each time he/she perceives the monofilament and to indicate which foot, “right or left,” the perception is on. The application must be repeated three times on the same site, one of which is a dummy. In the event of 2 out of 3 errors on a site, the patient is at risk of asymptomatic ulceration due to loss of protective sensitivity [15].

The diagnosis of DPN was affirmed in the presence of symmetrical distal sensory symptoms manifesting initially in the lower limbs with a DN4 questionnaire score of ≥4/10, impaired foot sensitivity as evidenced by a 10 g monofilament examination, and reduced or absent ankle reflexes [1, 16]. The diagnosis of retinopathy was made subsequent to a thorough examination of the vitreous and fundus by an experienced ophthalmologist, which revealed abnormalities [16]. The diagnosis of nephropathy was confirmed by the presence of albuminuria (≥30 mg/24 h) and/or reduced estimated glomerular filtration rate (<60 mL/min/1.73 m²) in the absence of signs or symptoms of other primary causes of kidney damage, associated with long‐standing diabetes, with or without diabetic retinopathy [17, 18].

### 2.4 Statistical Analysis

The data were collected via the patients' medical records. This information was entered into the Microsoft Excel spreadsheet, then imported and analyzed using IBM SPSS® software version 26.0. In this study, variables are expressed as percentages and medians with an interquartile range (IQR) between the 25th and 75th percentiles. To determine the risk factors for DPN, a statistical analysis was performed between DPN and the different variables. This analysis was based on Pearson's Chi‐square test, Fisher's exact test and odds ratio (OR) with a 95% confidence interval (95% CI). The statistical significance was set at the 5% confidence level with *p*‐value of <0.05.

### 2.5 Ethics Considerations

Prior to conducting the study, requests for authorization to collect data were submitted to the hospital's General Manager and the head of the department. The anonymity and confidentiality of the subjects under study were respected at all times. All patients participating in the study had provided written consent and had signed an informed consent form. The study was approved by the Review Board of the Soavinandriana hospital center on October 28, 2021.

## 3 Results

Among the 302 patients with T2DM who consented to participate in the study, 289 were included, and 13 were excluded, resulting in a nonresponse rate of 4.3%. The majority of patients (68.5%) were aged 60 years or over, with a median age of 64 (IQR 57–69). The male gender was observed in 51.6% of cases. The predominant cardiovascular risk factors were identified as dyslipidemia (78.5%) and arterial hypertension (70.6%). It should be noted that a patient may exhibit one or more cardiovascular risk factors. The median duration of diabetes was found to be 6 years (IQR 3–8), and it was already diagnosed in 79.2% of cases. The median glycated hemoglobin level was 8.9% (IQR 7.6–10.5). Oral antidiabetic medications were previously administered alone and in combination with insulin in 49.1% and 16.6% of cases, respectively (see Table 1).

**Table 1:** Distribution of patients according to general characteristics.

Variables	Categories	Effective	Percentage
Age group	[37–39 years]	10	3.5
	[40–49 years]	18	6.2
	[50–59 years]	63	21.8
	≥ 60 years	198	68.5
Sex	Male	149	51.6
	Female	140	48.4
Cardiovascular risk factors	Dyslipidemia	227	78.5
	Hypertension	204	70.6
	Menopause	114	39.4
	Microalbuminuria	89	30.8
	Current smoking	76	26.3
	Overweight or obesity	35	12.1
T2DM newly diagnosis	Yes	60	20.8
T2DM duration	[1–4 years]	94	32.5
	[5–9 years]	85	29.4
	[≥ 10 years]	50	17.3
Glycated hemoglobin	≥ 7%	241	83.4
	< 7%	48	16.6
Microvascular complications	Nephropathy	73	25.3
	Retinopathy	58	20.1
Antidiabetics medication	OAD	142	49.1
	Insulin	69	23.9
	OAD + insulin	48	16.6
	CLM	30	10.4
Antihypertensives medication	RAAS blockers	192	66.4
	CC blockers	112	38.8
	Diuretics	68	23.5
	Beta‐blockers	42	14.5
Statins	Yes	187	64.7
Antiplatelet agents	Yes	136	47.1

Abbreviations: CC, Calcium‐channel; CLM, Comprehensive lifestyle modification; OAD, Oral antidiabetics; RAAS, Renin‐angiotensin‐aldosterone system; T2DM, Type 2 diabetes mellitus.

The neuropathic pain was predominantly characterized by tingling (28.7%) and numbness (23.2%), with a DN4 questionnaire score greater than or equal to 4 observed in 19.7% of cases. A quarter of patients had an abnormal monofilament test and reduced or absent ankle reflexes. The DPN prevalence was 28.7% (see Table 2). All patients diagnosed with DPN received vitamin B supplementation, and 77 patients (26.6%) received gabapentin or pregabalin.

**Table 2:** Distribution of patients according to diabetic peripheral neuropathy.

Variables	Categories	Effective	Percentage
Symptoms	Tingling	83	28.7
	Numbness	67	23.2
	Burning	64	22.1
	Pins and needles	63	21.8
	Electric shocks	49	17.0
DN4 questionnaire	< 4	232	80.3
	≥ 4	57	19.7
Foot deformity	No	219	75.8
	Yes	70	24.2
Monofilament testing	Normal	219	74.7
	Reduced/absent	73	25.3
Ankle reflexes	Present	217	75.1
	Reduced/absent	72	24.9
DPN	No	206	71.3
	Yes	83	28.7

Abbreviation: DPN, diabetic peripheral neuropathy.

The risk factors for DPN were age ≥60 years, dyslipidemia, microalbuminuria, DM duration ≥ 10 years, Hb A1c ≥ 7%, nephropathy and retinopathy (see Table 3).

**Table 3:** Risk factors for diabetic peripheral neuropathy.

Variables	DPN		Bivariate analysis		Multivariate analysis	
	No (*n*1 = 206)	Yes (*n*2 = 83)	cOR (95% CI)	*p*‐Value	aOR (95% CI)	*p*‐Value
Male sex, *n* (%)	109 (52.9)	40 (48.2)	0.83 (0.48–1.42)	0.2754		
Age ≥ 60 years, *n* (%)	132 (64.1)	66 (79.5)	2.17 (1.15–4.25)	0.0048*	2.21 (1.19–4.08)	0.0111*
Dyslipidemia, *n* (%)	152 (73.8)	75 (90.4)	3.32 (1.47–8.49)	0.0006*	3.38 (1.52–7.52)	0.0028*
Hypertension, *n* (%)	142 (68.9)	62 (74.7)	1.33 (0.72–2.49)	0.2038		
Menopause, *n* (%)	77 (37.4)	37 (44.6)	1.35 (0.77–2.33)	0.1586		
Microalbuminuria, *n* (%)	38 (18.4)	51 (61.4)	6.98 (3.85–12.8)	< 0.0001*	6.55 (3.69–11.6)	< 0.0001*
Current smoking, *n* (%)	49 (23.8)	27 (32.5)	1.54 (0.84–2.79)	0.0849		
Overweight or obesity, *n* (%)	22 (10.7)	13 (15.7)	1.55 (0.68–3.42)	0.1642		
DM duration ≥ 10 years, n (%)	26 (12.6)	24 (28.9)	2.80 (1.42–5.52)	0.0007*	2.92 (1.54–5.53)	0.0010*
Hb A1c ≥ 7%, *n* (%)	166 (80.6)	75 (90.4)	2.25 (1.01–5.85)	0.0287*	2.43 (1.08–5.49)	0.0323*
Nephropathy, *n* (%)	22 (10.7)	51 (61.4)	13.1 (6.82–26.2)	< 0.0001*	12.8 (6.71–24.3)	< 0.0001*
Retinopathy, *n* (%)	31 (15.0)	27 (32.5)	2.71 (1.42–5.15)	0.0006*	2.32 (1.25–4.31)	0.0075*

Abbreviations: aOR, adjusted odds ratio by age; CI, Confidence interval; cOR, crude odds ratio; DM, diabetes mellitus; DPN, Diabetic peripheral neuropathy; Hb A1c, Glycated hemoglobin.

* Significant *p*‐value < 0.05.

## 4 Discussion

The prevalence of PND in the present study was similar to that observed in the Ugandan [19], European [20], and Qatari [21] studies, which were 29.4%, 25.2%, and 23.9%, respectively. However, several studies conducted in Ethiopia had found different prevalences of 14.3% [22], 52.2% [23] and 53.6% [24]. In a Ghanaian study, 20.6% of patients were found to have DPN based on abnormal perception of the vibratory test, and 35.6% based on the presence of clinical symptoms [25]. In Tanzania, the prevalence of DPN was as high as 72.2% [26]. Based on the Michigan Neuropathy Screening Instrument score, it was 39.5% in Jordan [27]. This discrepancy is influenced by variations in study populations across different regions and countries, particularly in the methods of diagnosing DPN, as well as in the proportions of various DPN risk factors. In any event, DPN is prevalent that merits particular consideration. In this context, the clinical interview remains the key point in the process of screening.

The DPN is most often manifested by neuropathic pain, which is characterized by the DN4 questionnaire, both in the present study and in a South African study [28]. A comprehensive, bilateral, and comparative physical examination of the feet should be systematically performed on all diabetic patients during each consultation. A study conducted in Jordan revealed that foot deformities were observed in 74% of cases, abnormal ankle reflexes were observed in 51% of cases, abnormal vibratory perception was observed in 66% of cases, and the monofilament test was abnormal in 78% of cases [27]. Furthermore, innovative point‐of‐care devices such as DPNCheck®, Neuropad®, and Sudoscan® have the potential to serve as objective screening tests for DPN. These are rapid, non‐invasive tests with acceptable sensitivity levels [29].

In the present study, age ≥ 60 years, dyslipidemia, microalbuminuria, DM duration ≥ 10 years, Hb A1c ≥ 7%, nephropathy and retinopathy were identified as risk factors for DPN. Similarly, in African studies, diabetics aged over 60 years were five‐ to sixfold increased risk of developing DPN [19, 22, 26]. Research conducted in developed countries has also demonstrated significant associations between advanced age and DPN, as in Qatar [21] and China [30]. Indeed, aging exerts a direct effect on metabolic regulation, which interacts with diabetes to accelerate the progression of many complications, including DPN [31]. Dyslipidemia has been identified as an independent risk factor for DPN [21, 27]. The mechanisms involved are hypothesized to be oxidative stress, inflammation, and direct lipotoxicity to neurons and Schwann cells [32]. In Cameroon, albuminuria was strongly associated with DPN, with an adjusted OR of 20.4 (6.5–63.9) [33]. A study conducted in Taiwan showed that an elevated albuminuria‐creatinuria ratio was associated with an increased risk of developing DPN [34]. As in several studies, a diabetes duration exceeding 10 years at least doubled the risk of DPN [21, 23, 27]. Additionally, Mekuria Negussie and Tilahun Bekele in Ethiopia showed that a diabetes duration >5 years was already a risk factor for DPN [22]. In a study conducted in China, glycated hemoglobin in diabetics with DPN was significantly higher than that without DPN [30]. Kuate‐Tegueu et al. in Cameroon also found that glycated hemoglobin >7.5% significantly increased the risk of DPN ninefold [33]. Indeed, DPN has an intricate pathophysiology involving glucotoxicity, advanced glycation end products and inflammatory and immune processes [35]. Among the various complications of diabetes, several authors have demonstrated significant associations between diabetic kidney disease, diabetic retinopathy and DPN [27, 33, 36].

However, the role of gender in the incidence of DPN differed from one study to another [20, 37]. Several studies have found that hypertensive diabetic patients were at least twice as likely to develop DPN [19, 22, 36]. In Ethiopian studies, overweight, obesity and smoking significantly increased the risk of DPN [23, 24].

The present study highlights the extent of DPN in Malagasy diabetics patients. It will also assist in the consolidation of the country's databases, and lead to an enhancement in the management of this complication. This study calls for the initiation of a regional and national research scale in this subject. Nevertheless, the monocentric nature of the study and its cross‐sectional study design have the potential to limit the generalizability of the results to the entire diabetic population and the identification of risk factors. The phenomenon of recruitment bias is another potential consideration in this context. It was noted that some data were absent from the patient records, including socioeconomic status, education level, physical activity level, and DPN biomarkers.

## 5 Conclusion

In conclusion, the prevalence of DPN was found to be significant among Malagasy patients with T2DM. The identified risk factors were consistent with those reported in the extant literature. Screening for DPN should be considered in elderly subjects, those with other cardiovascular risk factors, and those with long‐duration, uncontrolled diabetes complicated by other microangiopathies. Overall management must be early, appropriate and multidisciplinary. Achieving optimal glycemic control and controlling modifiable cardiovascular risk factors are paramount.

## Author Contributions

**Sitraka Angelo Raharinavalona:** conceptualization, investigation, writing – original draft, methodology, visualization, validation, writing – review and editing, software, formal analysis, project administration, data curation, resources. **Naliniaina Robert Randrianatoandro:** investigation, data curation, writing – review and editing. **Rija Eric Raherison:** validation, writing – review and editing. **Thierry Razanamparany:** data curation, writing – review and editing. **Andrianirina Dave Patrick Rakotomalala:** validation, writing – review and editing, supervision. All authors have read and approved the final version of the manuscript.

## Conflicts of Interest

The authors declare no conflicts of interest.

## Transparency Statement

The lead author, Sitraka Angelo Raharinavalona, affirms that this manuscript is an honest, accurate, and transparent account of the study being reported; that no important aspects of the study have been omitted; and that any discrepancies from the study as planned (and, if relevant, registered) have been explained.

## Data Availability

The datasets used and/or analyzed during the current study are available from the corresponding author upon reasonable request.
